# Profibrotic mediators in tendon disease: a systematic review

**DOI:** 10.1186/s13075-016-1165-0

**Published:** 2016-11-18

**Authors:** Wataru Morita, Sarah Jane Bothwell Snelling, Stephanie Georgina Dakin, Andrew Jonathan Carr

**Affiliations:** 1 Botnar Research Centre, Nuffield Department of Orthopaedics, Rheumatology and Musculoskeletal Sciences, University of Oxford, Nuffield Orthopaedic Centre, Windmill Road, Headington, Oxford, OX3 7LD UK; 2 NIHR Oxford Biomedical Research Unit, Botnar Research Centre, University of Oxford, Windmill Road, Headington, Oxford, OX3 7LD UK

**Keywords:** Tendon, Tendinopathy, Tendon tear, Fibrosis, Transforming growth factor beta, Bone morphogenic protein, Connective tissue growth factor

## Abstract

**Background:**

Tendon disease is characterized by the development of fibrosis. Transforming growth factor beta (TGF-β), bone morphogenic proteins (BMPs) and connective tissue growth factor (CTGF) are key mediators in the pathogenesis of fibrotic disorders. The aim of this systematic review was to investigate the evidence for the expression of TGF-β, BMPs and CTGF along tendon disease progression and the response of tendon cells to these growth factors accordingly.

**Method:**

We conducted a systematic screen of the scientific literature using the Medline database. The search terms used were “tendon AND TGF-β,” “tendon AND BMP” or “tendon AND CTGF.” Studies of human samples, animal tendon injury and overuse models were included.

**Results:**

Thirty-three studies were included. In eight studies the expression of TGF-β, BMPs or CTGF was dysregulated in chronic tendinopathy and tendon tear patient tissues in comparison with healthy control tissues. The expression of TGF-β, BMPs and CTGF was increased and showed temporal changes in expression in tendon tissues from animal injury or overuse models compared with the healthy control (23 studies), but the pattern of upregulation was inconsistent between growth factors and also the type of animal model. No study investigated the differences in the effect of TGF-β, BMPs or CTGF treatment between patient-derived cells from healthy and diseased tendon tissues. Tendon cells derived from animal models of tendon injury showed increased expression of extracellular matrix protein genes and increased cell signaling response to TGF-β and BMP treatments compared with the control cells (two studies).

**Conclusion:**

The expression of TGF-β, BMPs and CTGF in tendon tissues is altered temporally during healing in animal models of tendon injury or overuse, but the transition during the development of human tendon disease is currently unknown. Findings from this systematic review suggest a potential and compelling role for TGF-β, BMPs and CTGF in tendon disease; however, there is a paucity of studies analyzing their expression and stimulated cellular response in well-phenotyped human samples. Future work should investigate the dynamic expression of these fibrotic growth factors and their interaction with tendon cells using patient samples at different stages of human tendon disease.

**Electronic supplementary material:**

The online version of this article (doi:10.1186/s13075-016-1165-0) contains supplementary material, which is available to authorized users.

## Background

Tendon diseases are increasingly common fibrotic disorders [1, 2] and account for a third of all musculoskeletal complaints [3]. The patella, Achilles and rotator cuff (RC) tendons are the most frequently affected sites [4]. Tendon disorders are generally described by the term “tendinopathy” that includes diseases of the tendon and also tendon–bone junctions, namely enthesopathy or enthesitis [5]. The development of tendon disease has been proposed to start from an acute reactive tendinopathy, and subsequent dysregulated healing or disrepair may cause progression to chronic tendinopathy [6, 7]. The etiology is multifactorial, with overuse, trauma, aging and genetic predisposition regarded as notable risk factors [8, 9]. Recent studies have indicated the key role of inflammation in the homeostasis and healing of tendon, and its dysregulation may therefore contribute to the accumulation of mechanically inferior fibrotic tendon tissue [10, 11]. This results in an inappropriate function of the tendon, higher risk of reinjury [12] and tendon rupture [6]. Torn tendons are usually treated by surgical repair, but the postoperative retear rate remains high at around 40 % in operated RC tears [13]. A clinical need therefore exists for improved understanding of the mechanisms underlying both tendon disease and successful repair.

Studies of fibrotic diseases in other organs such as the liver, lung and kidney have implicated the members of the transforming growth factor beta (TGF-β) superfamily as key fibrotic growth factors [14] and proposed them as effective biomarkers for fibrotic changes [15]. TGF-β and bone morphogenic proteins (BMPs) are the two major members of the superfamily, the former known as a key regulator of fibrosis and repair. BMPs also contribute to fibrosis by guiding cell differentiation and the subsequent synthesis of extracellular matrix (ECM) proteins. TGF-β and BMPs both utilize the canonical Smad cell-signaling pathway, which also has been suggested to have crosstalk between the two [16]. Connective tissue growth factor (CTGF) belongs to the CCN family (CTGF, cysteine rich protein (Cyr61) and nephroblastoma overexpressed gene (Nov)) but is one of the key profibrotic mediators and acts closely with TGF-β as its downstream mediator. The expression of these growth factors has been reported to change over the progression of fibrotic liver diseases from hepatitis, cirrhosis to carcinogenesis [17, 18], and the cellular response to these fibrotic mediators differs accordingly [19].

The aim of this study was to investigate the role of TGF-β, BMPs and CTGF in the pathophysiology of tendon disease via a systematic review. The first objective was to investigate the gene and protein expression of TGF-β, BMPs and CTGF in diseased tendons along the development of disease from tendinopathy to tear in comparison with the healthy tendon tissues. The second objective was to investigate whether cellular response to treatment with TGF-β, BMPs or CTGF was dysregulated in tendon cells from diseased tendons. Animal studies of tendon injury and overuse models were also reviewed. We hypothesized that the expression of TGF-β, BMPs and CTGF in diseased or injured tendon tissues and the cellular responses to these growth factors would change between normal tendon, early and late disease or healing. Understanding disease progression in relation to the expression and cellular activity of these key fibrotic mediators should help identify their potential role in the pathogenesis of tendon disease.

## Methods

### Search strategies

This systematic review was designed, undertaken and reported based on the Preferred Reporting Items for Systematic Reviews and Meta-Analyses (PRISMA) Statement and the Cochrane guidelines. The inclusion criteria and analysis methods were defined and stated in a protocol prior to the study. Scientific literature was obtained using the Medline electronic database. The search was conducted in September 2016 with the following search terms: “tendon AND TGF-β,” “tendon AND BMP” and “tendon AND CTGF.” The reports retrieved by the searches were compiled and duplicates were removed. The abstract of the papers was screened before the assessment for eligibility by reviewing the full text of the articles.

The studies reporting the expression of the growth factors in diseased human tendon tissues, animal models of tendon injury or overuse had to relate to the mid-substance of tendon or tendon-to-bone enthesis, and therefore studies on muscle–tendon junctions, ligament reconstruction using tendon grafts and other soft tissues (e.g. muscles, ligaments, bursa and synovial tissues) were excluded. The included studies had to involve a control group. The animal studies of injury healing had to follow and report the temporal course of repair in comparison with the control. Animal overuse models were included when the development of tendinopathy was verified based on histological findings such as infiltration of inflammatory cells, changes in cellularity/cell phenotype, vascularity and disorganized or ruptured collagen fibers [5, 20]. The in vitro studies had to relate cell phenotype depending on the presence of disease to the cellular activity in response to treatment by TGF-β, BMPs or CTGF. Review articles, case reports and studies that were not reported in English were excluded. There was no limitation in the year of data entry, although all of the results were published after 1985.

The data extracted were summarized using a spreadsheet that included patient or animal model characteristics, method of tissue or cell analysis, the control group, results and statistical methods.

### Study selection

**Fig. 1:**
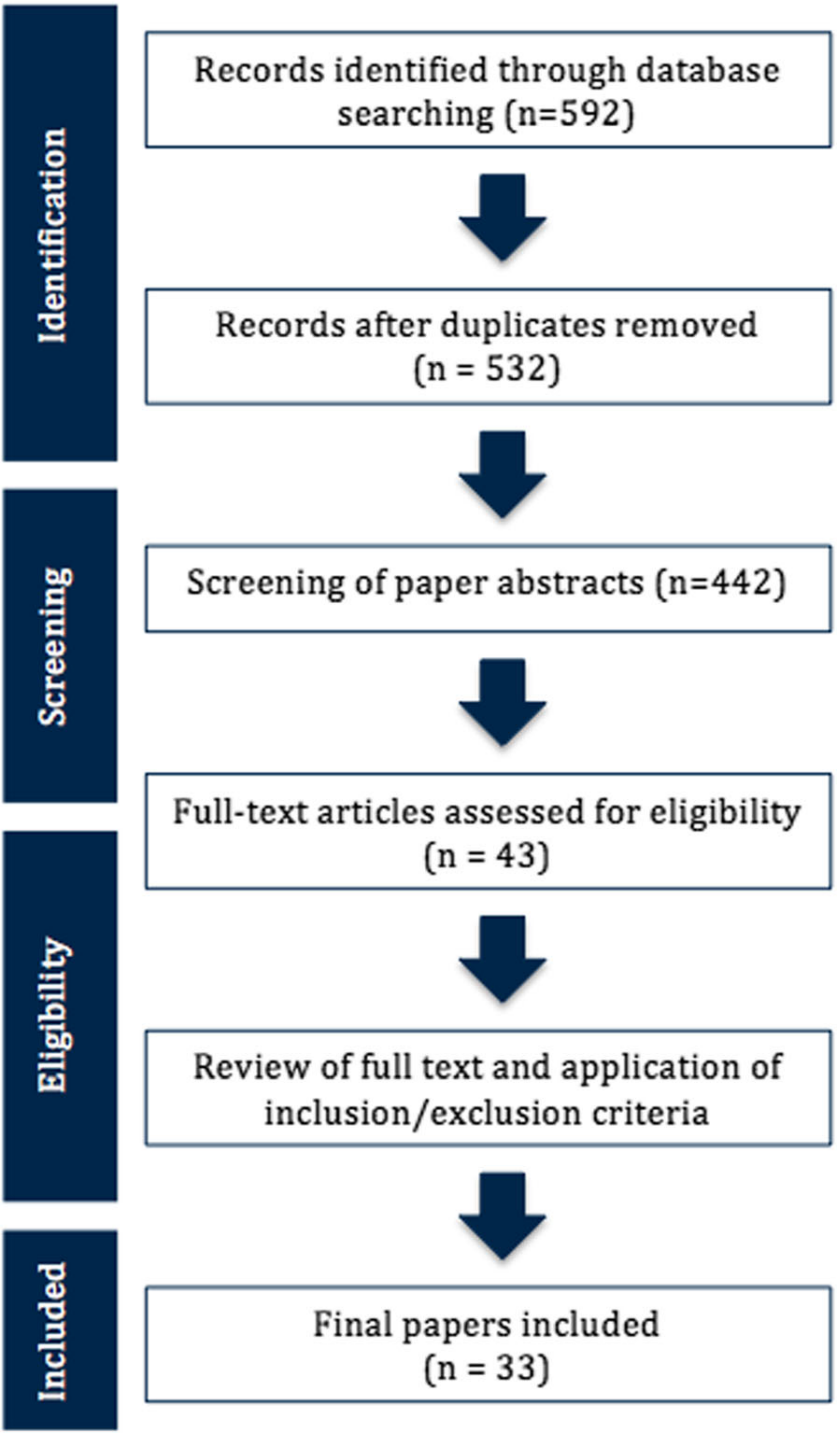
Flow chart of the systematic review protocol

The search yielded 592 results (Fig. 1). There were 532 papers after duplicates were removed, and 442 papers remained after review articles, case reports and articles that were not in English were removed. Screening of the paper abstracts based on the criteria set beforehand reduced this number to 43. Assessment for eligibility through the full text resulted in 33 papers meeting the criteria. The reasons for excluding the remaining 10 papers were no control group (*n* = 3), no temporal comparison of the expression of the growth factors in animal studies (*n* = 4) and no verification of the development of tendinopathy in an animal overuse model (*n* = 3). The papers that met the inclusion criteria are summarized in Tables 1, 2 and 3.

**Table 1:** Main characteristics of studies and patients of the papers included for expression in the diseased human tendon tissues search

Author	Year	Journal	Tendon	Healthy control?	Quantitative/semiquantitative analysis and statistical tests	Investigated genes and proteins
TGF-β						
Goodier et al. [22]	2016	*Arthritis Res Ther*	Rotator cuff	Yes	Yes	TGFβ1, TGFβR1, TGFβR2
Pingel et al. [25]	2013	*Eur J Appl Physiol*	Achilles	Yes^a^	Yes	TGFβ1
Pingel et al. [27]	2012	*BMC Musculoskeletal Disord*	Achilles	Yes^a^	Yes	TGFβ1
de Mos et al. [28]	2009	*Am J Sports Med*	Achilles	Yes	Yes	TGFβ
Fu et al. [24]	2002	*Clin Orthop Relat Res*	Patella	Yes	Yes	TGFβ1
Fenwick et al. [23]	2001	*J Anat*	Achilles	Yes^b^	Yes	TGFβ1, TGFβ2, TGFβ3, TGβR1, TGβR2
BMPs						
Goodier et al. [22]	2016	*Arthritis Res Ther*	Rotator cuff	Yes	Yes	BMP2, BMP7
Rui et al. [26]	2012	*Knee Surg Sports Traumatol Arthrosc*	Patella	Yes	No	BMP2, BMP4, BMP7
Oliva et al. [29]	2011	*Eur Cell Mater*	Rotator cuff	Yes^a^	Yes	BMP2, BMP4, BMP6
CTGF						
Goodier et al. [22]	2016	*Arthritis Res Ther*	Rotator cuff	Yes	Yes	CTGF
Pingel et al. [27]	2012	*BMC Musculoskeletal Disord*	Achilles	Yes^a^	Yes	CTGF

^a^Studies that sampled control tissues from the macroscopically healthy region of the same tendon

^b^Studies that sampled control tissues from cadavers

*BMP* bone morphogenic protein, *CTGF* connective tissue growth factor, *TGF-β* transforming growth factor beta

**Table 2:** Main characteristics of studies and animal models of the papers included for expression in the tendon injury or overuse model tissues search

Author	Year	Journal	Tendon	Model	Healthy control?	Quantitative/semiquantitative analysis and statistical tests	Investigated genes and proteins
TGF-β							
Zhang et al. [47]	2016	*Matrix Biol*	Achilles, mouse	Transection	Yes	Yes	TGFβ1, TGFβ2, TGFβ3, TGFβR1, TGFβR2
Gao et al. [30]	2013	*PLoS One*	FD, rat	Overuse	Yes	Yes	TGFβ1
Heisterbach et al. [48]	2012	*Knee Surg Sports Traumatol Arthrosc*	Achilles, rat	Transection → repair	Yes	Yes	TGFβ1
Otoshi et al. [49]	2011	*Arthroscopy*	Achilles, rat	Removal	Yes	Yes	TGFβ1
Lin et al. [50]	2010	*Bone*	Achilles, rat	Transection	Yes	Yes	TGFβ1, TGFβ2, TGFβ3
Chen et al. [51]	2008	*J Hand Surg Am*	FDP, chicken	Transection → repair	Yes	Yes	TGFβ
Chan et al. [52]	2008	*Wound Repair Regen*	Patella, rat	Defect	Yes	Yes	TGFβ1, TGFβ2, TGFβ3, TGFβR1, TGFβR2
Würgler-Hauri et al. [53]	2007	*J Shoulder Elbow Surg*	RC, rat	Detach → repair	Yes	No	TGFβ1
Berglund et al. [54]	2006	*J Hand Surg Am*	FDP, rabbit	Division → repair	Yes	Yes	TGFβ1
Kobayashi et al. [55]	2006	*J Shoulder Elbow Surg*	RC, rabbit	Defect	Yes	No	TGFβ
Galatz et al. [56]	2006	*J Orthop Res*	RC, rat	Transection → repair	Yes	Yes	TGFβ1, TGFβ3
Dahlgren et al. [57]	2005	*J Orthop Res*	FDS, horse	CI	Yes	No	TGFβ1
Darmani et al. [58]	2004	*Mediators Inflamm*	FD, rat	Crush injury	Yes	No	TGFβ
Ngo et al. [59]	2001	*Plast Reconstr Surg*	FDP, rabbit	Transection → repair	Yes	No	TGFβR1, TGFβR2, TGFβR3
Chang et al. [60]	1997	*Plast Reconstr Surg*	FDP, rabbit	Transection → repair	Yes	Yes	TGFβ1
Natsu-ume et al. [61]	1997	*J Orthop Res*	Patella, rat	Transection	Yes	Yes	TGFβ
BMPs							
Zhang et al. [47]	2016	*Matrix Biol*	Achilles, mouse	Transection	Yes	Yes	BMP1– BMP7, BMP12– BMP14, BMPR1, BMPR2
Heisterbach et al. [48]	2012	*Knee Surg Sports Traumatol Arthrosc*	Achilles, rat	Transection → repair	Yes	Yes	BMP12
Yee Lui et al. [62]	2011	*J Orthop Res*	Patella, rat	CI	Yes	Yes	BMP2, BMP4, BMP7
Lin et al. [50]	2010	*Bone*	Achilles, rat	Transection	Yes	Yes	BMP2, BMP4, BMP7
Lui et al. [63]	2009	*J Orthop Surg Res*	Patella, rat	CI/defect	Yes	Yes	BMP2
Eliasson et al. [64]	2008	*Clin Orthop Relat Res*	Achilles, rat	Transection	Yes	Yes	BMP7, BMP12, BMP13, BMP14, BMPR
Würgler-Hauri et al. [53]	2007	*J Shoulder Elbow Surg*	RC, rat	Detach → repair	Yes	No	BMP12, BMP13, BMP14
CTGF							
Gao et al. [30]	2013	*PLoS One*	FD, rat	Overuse	Yes	Yes	CTGF
Kietrys et al. [31]	2012	*PLoS One*	FD, rat	Overuse	Yes	Yes	CTGF
Fedorczyk et al. [32]	2010	*J Orthop Res*	FD, rat	Overuse	Yes	Yes	CTGF
Chen et al. [51]	2008	*J Hand Surg Am*	FDP, chicken	Transection	Yes	Yes	CTGF
Asundi et al. [42]	2008	*Eur J Appl Physiol*	FDP, rabbit	Overuse	Yes	Yes	CTGF
Würgler-Hauri et al. [53]	2007	*J Shoulder Elbow Surg*	RC, rat	Detach → repair	Yes	No	CTGF
Berglund et al. [54]	2006	*J Hand Surg Am*	FDP, rabbit	Division → repair	Yes	Yes	CTGF
Nakama et al. [43]	2006	*J Orthop Res*	FDP, rabbit	Overuse	Yes	Yes	CTGF

*BMP* bone morphogenic protein, *CI* collagenase-induced, *CTGF* connective tissue growth factor, *FD* flexor digitorum, *FDP* flexor digitorum profundus, *FDS* flexor digitorum superficialis, *RC* rotator cuff, *TGF-β* transforming growth factor beta

**Table 3:** Main characteristics of studies and cells of the papers included for the cellular responses to treatment search

Author	Year	Journal	Tendon	Model	Healthy control?	Quantitative/semiquantitative analysis and statistical tests	Treatment
TGF-β							
Fu et al. [34]	2008	*J Orthop Res*	Patella, rat	Defect	Yes	Yes	TGFβ1
BMPs							
Lui and Wong [33]	2013	*BMC Musculoskeletal Disord*	Patella, rat	CI	Yes	Yes	BMP2

*BMP* bone morphogenic protein, *CI* collagenase-induced, *TGF-β* transforming growth factor beta

### Study characteristics

Only one study compared the expression of TGF-β, BMPs and CTGF between different stages of human tendon disease in the RC. Seven studies compared the differences in the expression of at least one of the growth factors between tendinopathic and healthy tendon tissues in the patella, Achilles or RC (Table 1). However, the tissue was sampled from calcific tendinopathy patients in one study and another study compared the expression of the proteins after an intervention by exercise. Sixteen, seven and eight studies respectively reported the temporal expression of TGF-β, BMPs and CTGF in animal models of tendon injury or overuse (Table 2). The injury models varied by using the patella, Achilles, RC or flexor digitorum (FD) tendons in mice, rats, chickens, rabbits or horses, with injuries or defects created by transection, crush injury, use of collagenase or longitudinal division. Whether the transected tendons were repaired surgically or spontaneous healing was observed depended on the study. The development of tendinopathy was verified by histology in the overuse models, which used the RC or FD tendons in rats or rabbits. No study compared the differences in the cellular response to TGF-β, BMPs or CTGF in healthy and diseased cells from human tendons. One study used rat tendon cells from the patella of a collagenase-induced (CI) tendon injury model and another from a rat patella defect model, and treated the cells in vitro with BMP2 and TGF-β, respectively (Table 3). The cellular response to these growth factors was measured by the cell proliferation rate, expression of ECM-related genes and phosphorylation level of the Smad proteins, which are the key mediators of the TGF-β and BMP cell-signaling pathway.

### Study methodology and assessing the risk of bias

The quality of study methodology was assessed by referring to the modified scoring system by Dean et al. [21] in order to highlight the studies with a high risk of potential bias (Additional file 1). The mean (standard deviation) score was 7.73 (1.28) out of 10. All included studies had a control group, although in two studies the “control” samples were obtained from a macroscopically healthy area of the same tendon that may be at a risk of bias. Ten animal studies did not clarify the age or sex of the animals used. The methods of tissue and cell sampling and analysis were clearly described in all studies. Seven studies did not use quantitative measures or statistical analysis for comparison. The data were checked for normality in only four studies. All except 11 studies stated the set statistical significance. Two studies set *p* < 0.01 with consideration for an increased type I error by performing multiple tests, but the remaining studies set *p* < 0.05. The limitations of the study were not addressed in 13 studies. Meta-analysis was not performed due to the heterogeneity of the identified studies.

## Results

### Expression of TGF-β, BMPs and CTGF in diseased human tendon tissues

The expression of TGF-β, BMPs and CTGF was dysregulated at different stages of tendon disease (Table 4); the single study that compared the protein expression of these growth factors between torn, tendinopathic and healthy RC tissues reported a decreased expression of TGF-β and its receptors in the diseased tendon tissues of both chronic tendinopathy and tear [22]. Gene and protein expression of TGF-β and protein expression of BMP2, BMP4 and BMP7 were increased in the six studies that compared tendinopathy and healthy tendon tissues from the patella or the Achilles [23–28]. The gene expression of BMP4 and BMP6 was suppressed in the calcific area of calcific tendinopathy of the RC [29]. The diagnosis of chronic tendinopathy was made clinically in all seven studies of human disease, with auxiliary imaging methods such as MRI or US [22–28]. The duration of pain was defined as more than 3 and 6 months in one study [28] and in four studies [22, 25–27] respectively, but there was no clear explanation in the other two studies [23, 24]. The control tissues were obtained from the same anatomical location except in one study that investigated the gene expression in RC tear compared with the hamstring tendons in patients going under anterior cruciate ligament reconstruction surgery [22]. In the other studies that utilized the same anatomy, two studies obtained the control tissues from the unaffected area diagnosed by US in the same tendon [25, 27], and one study used cadaveric samples [23]. One study focused on a particular subtype of tendinopathy, calcific tendinopathy [29]. The two studies that investigated the gene expression of CTGF in RC tendon tear tissues did not show significant differences compared with the healthy tendon tissues [22, 27]. The studies investigating the effect of exercise in human tendons were excluded because the primary objective was to investigate the expression pattern across the spectrum of tendon disease and not to assess the impact of activity in tendons.

**Table 4:** Expression of TGF-β, BMPs and CTGF in diseased human tendon samples versus healthy control tendon

	Tendon	Increased, unchanged, decreased in diseased vs control	
		Gene	Protein
Tendinopathy			
TGFβ	Achilles	↑ [29]	
TGFβ1	RC, Achilles, patella	↑ [25, 27]	↓ [22], ↑ [24], → [23]
TGFβ2	Achilles		↑ [23]
TGFβ3	Achilles		→ [23]
TGFβR1	RC, Achilles		→ [22, 23]
TGFβR2	RC, Achilles		↓ [22, 23]
BMP2	RC, patella	→ [29]	↑ [26]
BMP4	RC, patella	↓ [29]	↑ [26]
BMP6	RC	↓ [29]	
BMP7	Patella		↑ [26]
CTGF	Achilles	→ [27], ↓ [25]	
Tear			
TGFβ1	RC	→ [22]	↓ [22]
TGFβR1	RC	↓ [22]	↓ [22]
TGFβR2	RC	↑ [22]	↓ [22]
BMP2	RC	→ [22]	
BMP7	RC	→ [22]	
CTGF	RC	→ [22]	

*BMP* bone morphogenic protein, *CTGF* connective tissue growth factor, *RC* rotator cuff, *TGF-β* transforming growth factor beta

### Expression of TGF-β, BMPs and CTGF in animal models of tendon injury or overuse

There was a wide variety of animal injury models, and the gene and protein expression of TGF-β was predominantly increased in healing compared with healthy tendon tissues. However, the temporal pattern of the transition was inconsistent (Table 5). The expression of BMPs and CTGF was variable and could be increased, decreased or similar to that of the healthy tissues. In the overuse models, the expression of TGF-β1 and CTGF proteins did not show changes in the early stages of intervention but increased after 3 months [30–32] (Table 5). No animal studies of tendon overuse focused on the expression of BMPs (Table 5).

**Table 5:** Expression of TGF-β, BMPs and CTGF in animal models of tendon injury or overuse versus healthy control tendon

	Animal	Tendon and model	Increased, unchanged, decreased in animal models vs control	
			Gene	Protein
Injury models				
TGFβ	Rat	Patella, transection (partial)	↑ (1, 3, 7, 14, 28, 56 d) [61]	
	Chicken	FDP, transection → repair	↑ (3 d), → (9, 14, 21 d) [51]	
TGFβ1	Rat	Achilles, transection		→ (2, 4, 6, 8 w) [48]
		Achilles, removal		→ (2 d), ↑ (7 d), → (30, 90, 180 d) [49]
	Mouse	Achilles, transection	↑ (1, 2, 4 w) [47]	
	Rabbit	FDP, division → repair	↑ (3, 6, 12 d), → (24 d) [54]	
		FDP, transection → repair	→ (1 d), ↑ (3, 7, 14, 28, 56 d) [60]	
	Horse	FDS, CI	↑ (1, 2, 4 w), → (8 w), ↑ (24 w) [57]	→ (1, 2 w), ↑ (4, 8 w), → (24 w) [57]
TGFβ2	Rat	Achilles, transection	↑ (3, 5, 10 w) [50]	↑ (3, 5, 10 w) [50]
	Mouse	Achilles, transection	↑ (1, 2, 4 w) [47]	
TGFβ3	Rat	Achilles, transection	↑ (3, 5, 10 w) [50]	↑ (3, 5, 10 w) [50]
	Mouse	Achilles, transection	→ (1 w), ↑ (2, 4 w) [47]	
TGFβR1	Mouse	Achilles, transection	→ (1 w), ↑ (2 w), → (4 w) [47]	
TGFβR2	Mouse	Achilles, transection	↑ (1, 2, 4 w) [47]	
TGFβR3	Mouse	Achilles, transection	→ (1, 2, 4 w) [47]	
BMP1	Mouse	Achilles, transection	↑ (1, 2, 4 w) [47]	
BMP2	Rat	Achilles, transection	↑ (3, 5, 10 w) [50]	↑ (3, 5, 10 w) [50]
		Patella, CI	↑ (2 w), → (4, 12 w) [63]	↑ (2, 4, 8, 12 w) [62, 63], ↑ (16 w) [62]
		Patella, defect	→ (2, 4, 12 w) [63]	↑ (2, 4, 12 w) [63]
	Mouse	Achilles, transection	↓ (1, 2, 4 w) [47]	
BMP3	Mouse	Achilles, transection	↑ (1, 2, 4 w) [47]	
BMP4	Rat	Achilles, transection	↑ (3, 5, 10 w) [50]	↑ (3, 5 w), → (10 w) [50]
		Patella, CI		↑ (2, 4, 8, 12, 16 w) [62]
	Mouse	Achilles, transection	→ (1 w), ↑ (2, 4 w) [47]	
BMP5	Mouse	Achilles, transection	→ (1 w), ↑ (2, 4 w) [47]	
BMP6	Mouse	Achilles, transection	→ (1, 2, 4 w) [47]	
BMP7 OP-1	Rat	Achilles, transection	↑ (3, 5, 10 w) [50]	↑ (3, 5, 10 w) [50]
		Achilles, transection → unload	↑ (3, 8 d), → (14, 21 d) [64]	
		Achilles, transection → load	→ (3, 8, 14 d), ↓ (14, 21 d) [64]	
		Patella, CI		↑ (2, 4, 8, 12, 16 w) [62]
	Mouse	Achilles, transection	→ (1 w), ↑ (2, 4 w) [47]	
BMP12	Rat	Achilles, transection		↑ (2 w), → (4, 6, 8 w) [48]
		Achilles, transection → unload	↓ (3, 8 d), → (14, 21 d) [64]	
		Achilles, transection → load	↓ (3, 8 d), → (14, 21 d) [64]	
	Mouse	Achilles, transection	→ (1 w), ↑ (2, 4 w) [47]	
BMP13	Rat	Achilles, transection → unload	→ (3, 8, 14 d), ↓ (21 d) [64]	
		Achilles, transection → load	→ (3, 8, 14 d), ↓ (21 d) [64]	
	Mouse	Achilles, transection	→ (1, 2, 4 w) [47]	
BMP14	Rat	Achilles, transection → unload	↓ (3, 8, 14, 21 d) [64]	
		Achilles, transection → load	↓ (3, 8, 14, 21 d) [64]	
	Mouse	Achilles, transection	↓ (1 w), ↑ (2 w), → (4 w) [47]	
BMPR1a	Mouse	Achilles, transection	↑ (1, 2, 4 w) [47]	
BMPR1b	Rat	Achilles, transection → unload	↓ (3 d), → (8 d), ↑ (14 d), ↓ (21 d) [64]	
		Achilles, transection → load	→ (3, 8, 14 d), ↓ (21 d) [64]	
	Mouse	Achilles, transection	→ (1 w), ↑ (2, 4 w) [47]	
BMPR2	Rat	Achilles, transection → unload	→ (3 d), ↑ (8, 14 d), ↓ (21 d) [64]	
		Achilles, transection → load	→ (3 d), ↑ (8, 14 d), ↓ (21 d) [64]	
	Mouse	Achilles, transection	↑ (1, 2, 4 w) [47]	
CTGF	Rabbit	FDP, division → repair	→ (3, 6, 12, 24 d) [54]	
	Chicken	FDP, transection → repair	↑ (3 d), → (9 d), ↓ (14, 21 d) [51]	
Overuse models				
TGFβ1	Rat	FD, hand-pulling task		→ (18 w), ↑ (24 w) [30]
CTGF	Rat	RC, hand-pulling task		↑ (12 w) [31]
		FD, hand-pulling task		↑ (18, 24 w) [30]
				→ (3, 6 w), ↑ (12 w) [32]
	Rabbit	FDP, electrical stimulation	→[42]	↑[43]

*BMP* bone morphogenic protein, *CI* collagenase-induced, *CTGF* connective tissue growth factor, *d* day(s), *FD* flexor digitorum, *FDP* flexor digitorum profundus, *FDS* flexor digitorum superficialis, *RC* rotator cuff, *TGF-β* transforming growth factor beta, *w* week(s)

### Response of tendon cells to treatment by TGF-β, BMPs or CTGF

There were no studies that investigated the differences in the cellular response to treatment with TGF-β, BMPs or CTGF by tendon cells from healthy and diseased human tendon tissues. Two studies used patella tendon derived cells from rat models of acute-stage tendon healing: one showed that diseased tendon cells from a CI tendon injury model had a higher cell signaling activity of the canonical Smad pathway in response to BMP stimulation compared with the healthy cells [33]; and the other reported that the expression of ECM genes such as collagens type I and III, decorin and biglycan to TGF-β treatment goes through temporal changes during tendon healing in a defect model [34] (Table 6).

**Table 6:** Cellular responses to treatment by TGF-β and BMPs in injured versus healthy tendon cells

		Increased, unchanged, decreased in diseased vs control	
Treatment	Cells	Gene	Protein
TGFβ1	Patella, rat, defect	Col1a1→, Col3a1↑, Dcn↑, Bgn → (day 7 healing) [34] Col1a1→, Col3a1→, Dcn↑, Bgn↓ (day 14 healing) [34]	
BMP2	Patella, rat, CI		p-Smad1/5/8↑ [33]

*BMP* bone morphogenic protein, *CI* collagenase-induced, *TGF-β* transforming growth factor beta

## Discussion

This study provides evidence for the involvement of TGF-β, BMPs and CTGF in the pathogenesis and healing of tendon disease. Diseased tissues from tendinopathy and tendon tear patients showed an inconsistent expression of these fibrotic growth factors that could be increased, decreased or similar to that of the healthy tissues. We were not able to identify a consistent temporal pattern in their expression along the development of the disease. Differences in the cellular responses to TGF-β, BMPs and CTGF in diseased compared with healthy human tendon cells had not been considered. However, animal studies of tendon injury healing showed a variety of expression patterns of these growth factors over time, and that the cellular activities of these factors also differ according to the phase of the healing.

Because of the heterogeneity of the included studies, we were not able to determine a specific role of TGF-β, BMPs or CTGF in the development of tendon disease but only suggest their involvement in the pathogenesis of fibrotic repair. The general applicability of the results is limited. Clinical samples were harvested from tendinopathy patients in all of the human studies, but the diagnosis criteria were nonuniform and the anatomical location varied, which included the patella, Achilles and RC. Gene expression and stimulatory effects of the pro-fibrotic growth factors on cells have been shown to differ by anatomical location in animal models [35, 36], and this is likely also the case for humans although we did not identify any studies assessing anatomical location in humans. The selection of matched control tissues with regards to clinical and ethical feasibility is another concern. In one study the control tissues were obtained from the hamstring tendons in patients going under anterior cruciate ligament reconstruction surgery in comparison with RC tear tendons [22], with an age difference between the two cohorts that could be a potential confounder in humans [37–39], unlike animals [40]. Whether an ultrasonographically intact region of a diseased tendon represents a healthy tissue remains controversial, and cadaveric tissue samples have their limitations in the traceability of tendon disease history and the effects of postmortem change [22].

For the studies of animal tendon injury models, only the studies that measured the temporal changes were included so that the results came in line with the primary objective of assessing differences between the stages of a tendon disease. Only the studies that used quantitative measures and statistical methods for comparison for their results were included (Table 5), but there was also a variation in the methods of analysis. Various animal models of tendinopathy have been proposed, but because they do not always account for the effects of aging and development of fibrosis, whether they accurately recapitulate human disease is open to debate [41]. We included the studies of overuse models that confirmed the development of tendinopathy after the intervention with (semi)quantitative methods such as changes in cellularity [30, 31], infiltration of inflammatory cells [32] and disorganized collagen fibers by histology [42, 43]. The expression of TGF-β1 and CTGF proteins increased after 3 months of overloading tasks corresponding to tendinopathy development, but not in the early stages of intervention [30–32].

We have indicated that the gene and protein expression of TGF-β and BMPs varies across the stage of tendon disease, and that cellular activities go through changes accordingly. This is in line with a previous study reporting the differences in the cellular response to TGF-β in human fibroblasts derived from normal skin and the skin of a hypertrophic scar [44]. Involvement of CTGF could not be established in human disease because only two studies looked at this growth factor [22, 25], and no difference in gene expression between healthy and diseased tendon was detected. There were also no studies that investigated differences in cellular activities in response to CTGF between cells from different stages of a tendon disease or injury healing. Further investigation is necessary to enhance our understanding of the role of TGF-β, BMPs and CTGF in tendon disease.

This review also highlights the paucity of analyses of human-derived tendon tissue and cells. The majority of studies to date have attempted to modify disease progression or improve healing by adjusting the expression of the fibrotic mediators that was found to be remarkable in diseased tendons, without a comprehensive understanding of their role and mechanisms in the development of the disease. Cells from clinical samples of tendinopathy show an altered phenotype [45] and the cellular activities of RC tear tendon cells may likewise differ according to the severity of the disease [46]. Despite these differential cellular responses, the majority of in vitro tendinopathy studies have not taken this into account. It is imperative to recognize that cultured tendon cells from intact as opposed to diseased tendons may not recapitulate the actual healing or disease development process with regards to cell proliferation and synthesis of ECM proteins [45]. Animal models have been beneficial in increasing the understanding of tendon disease progression and healing, but do not replicate the continuum of tendon disease [22]. It should be noted that cautious interpretation of animal studies is necessary. The most effective method to investigate the mechanism of human tendon pathophysiology is to use fresh well-phenotyped tissue from human patients with acceptable control samples. Understanding the interaction of cells and fibrotic mediators that drive fibrosis in the pathophysiology of tendon disease would be essential in order to identify the fundamental interventions, and therefore it is critical to appropriately define the details of the specimen such as origin of the tissues or cell, phase of the disease, criteria and method of diagnosis. In tendon disease, the importance of considering the appropriate disease phase and the corresponding intervention has been recognized [19]; however, the sequential regulation and interaction of the TGF-β, BMPs and CTGF with tendon cells is not yet well understood. Future work should investigate the temporal expression of these fibrotic growth factors and their interaction with tendon cells using well-phenotyped patient samples at different stages of tendon disease. This research will advance our knowledge of the pathophysiology of tendon disease, and will facilitate identification of potential therapeutic targets to enhance the quality of tissue repair or influence disease progression.

## Conclusions

The expression of the TGF-β, BMPs and CTGF is dysregulated in diseased human tendon tissues, but the transition during the development of the disease is yet to be defined. Cell behavior in response to these growth factors in human tendon cells along the course of pathology has not been explored. Importantly this review highlights the paucity of analyses of human-derived tendon tissues and cells. Further research using well-phenotyped patient samples at different stages of tendon disease is warranted to improve the understanding of disease pathogenesis.

## Additional file

Additional file 1: Presenting assessment of study methodology quality. The quality of methodology was assessed in the included 33 studies in order to highlight the reports with a high risk of potential bias. (XLS 41 kb)

## Abbreviations

BMP: Bone morphogenic protein
CI: Collagenase-induced
CTGF: Connective tissue growth factor
ECM: Extracellular matrix
FD: Flexor digitorum
PRISMA: Preferred Reporting Items for Systematic Reviews and Meta-analyses
RC: Rotator cuff
TGF-β: Transforming growth factor beta
